# An evidence mapping of systematic reviews and meta-analysis on traditional Chinese medicine for ulcerative colitis

**DOI:** 10.1186/s12906-021-03387-y

**Published:** 2021-09-13

**Authors:** Yu-Xin Sun, Xiao Wang, Xing Liao, Jing Guo, Wen-Bin Hou, Xin Wang, Jian-Ping Liu, Zhao-Lan Liu

**Affiliations:** 1 Center for Evidence-Based Chinese Medicine, Beijing University of Chinese Medicine, Beijing, 100029 China; 2 Center for Evidence Based Chinese Medicine, Institute of Basic Research in Clinical Medicine, China Academy of Chinese Medical Sciences, Beijing, 100010 China; 3 Neurology Department, China-Japan Friendship Hospital, Beijing, 100029 China; 4 Center for Studies in Constitution Research of Traditional Chinese Medicine, Beijing University of Chinese Medicine, Beijing, 100029 China

**Keywords:** Evidence-based medicine, Mapping review, AMSTAR 2, Herbal, Digestion

## Abstract

**Background:**

Traditional Chinese Medicine (TCM) has been a proposed treatment option for ulcerative colitis (UC), however it has been difficult to understand the breadth and depth of evidence as various Chinese medicine therapies may produce effects differently. The aim of this evidence mapping is to visually understand the available evidence in the use of TCM in the treatment of UC, and to identify gaps in evidence to inform priorities of future research.

**Methods:**

A systematic electronic literature search of six databases were performed to identify systematic reviews (SRs) on different Chinese medicine therapies in the treatment in UC. Methodological quality of the included SRs was assessed using AMSTAR 2.

**Results:**

The mapping was based on 73 SRs, which included nine interventions that met eligibility criteria. The quality of the included SRs was very low. The diseases stages of patients with UC varied greatly, from active to remission, to non-acute outbreak, to not reported. The results mostly favored the method of intervention. Oral administration combined with enema was the most widely used route of administration in secondary research.

**Conclusion:**

Based on the current evidence, the treatment of UC with TCM can only be recommended cautiously. A majority of included SRs did not report the location of the disease, the disease classification, and the route of administration of the intervention. Further research is needed on the effectiveness of Chinese medicine alone in the treatment of UC. The effectiveness of combined Chinese and conventional medicine combined with different routes of administration cannot be confirmed. Attention should be paid to the methodological quality of the systematic review. Unifies the outcome indicators used in the evaluation of effectiveness.

**Supplementary Information:**

The online version contains supplementary material available at 10.1186/s12906-021-03387-y.

## Background

Ulcerative colitis (UC) is a type of inflammatory bowel disease (IBD) caused by a variety of factors. UC has a tendency of recurrence throughout life [1, 2]. Crohn’s disease (CD) and UC are the main disease types [3]. In 1875, Wilks and Moxon established the term UC into the medical vernacular [4, 5]. The main clinical manifestations of this condition are recurrent diarrhea, mucus bloody stool, and abdominal pain [6]. The primary purpose of UC treatment is to control the acute onset of the disease, heal the mucosa, maintain remission, reduce recurrence, and prevent complications [7].

Several studies have reported that UC appeared initially in urban areas, where its incidence rose rapidly before decreasing slowly [8]. The crude annual overall incidence for IBD per 100,000 individuals in 2011–2012 was 1.37 in Asia. China have high disease incidence according to an inception cohort study [9]. The incidence of IBD in China has risen by threefold in the past decade. UC is the predominant IBD in Asia [10].

This evidence mapping is largely driven by the execution of SRs and meta-analysis. In recent years, evidence-based studies of TCM in the treatment of UC are continuing to increase. At present, there are two overviews of SRs [11, 12] about the treatment of UC with TCM. One focuses on treatment with retention-enema of Chinese herbal medicine, and the other uses an overview method to summarize the evidence. Our study is not limited to the route of administration in treatment; rather our focus is on the distribution of evidence. There is value in evaluating research conducted in terms of quality and interventions used to help evaluate progress made to date as well as determine future directions in research. We used evidence mapping to visualize the results, where the systematic review mapping can map out and categorize existing literature on a particular topic in order to identify gaps in research literature from which to commission further reviews and/or primary research [13].

Mapping review is used to present evidence in a field using a visual graph or chart after systematic researched. These maps provide assessments of knowledge gaps, knowledge gluts, and patterns across the research literature that promote best practice and direct research resources towards the highest quality research [14]. It can provide a broad and often comprehensive summation of a topic area and, as such, have value for those coming to a subject for the first time. However, as it is difficult to obtain a complete overview of a research topic for a single original study or a systematic review, we chose to conduct a mapping review of SRs. To provide the depth and width of current evidence on various interventions, we conducted an overview of relevant systematic reviews that have been published to date. Therefore, the objective of this study was to identify, describe and organize the current available evidence about TCM on the treatment of UC.

## Methods

### Criteria for considering reviews for inclusion

A comprehensive search of databases PubMed, Cochrane Library, CNKI, WanFang, VIP, and SinoMed, was conducted for systematic reviews published from database inception up to and including March 16, 2021. Search terms included “ulcerative colitis”, “Chinese herbal medicine”, “plant”, “systematic review” and “meta”.

Search strategy used in PubMed database:

*#1 Search (((ulcerative colitis[MeSH Terms]) OR ulcerative colitis[Title/Abstract]) OR ulcerative colitis[Text Word]) OR ulcerative colitis[Other Term].*

*#2 Search ((((((((((Traditional Chinese Medicine[Title/Abstract]) OR Traditional Medicine, Chinese[Title/Abstract]) OR Chinese Traditional Medicine[Title/Abstract]) OR Chinese Medicine, Traditional[Title/Abstract]) OR Phytotherapy[Title/Abstract]) OR Medicine, traditional[Title/Abstract]) OR Medicine, Chinese Traditional[Title/Abstract]) OR Plants, Medicinal[Title/Abstract]) OR Herbal Medicine[Title/Abstract]) OR Plant Preparations[Title/Abstract]) OR Drugs, chinese herbal[Title/Abstract].*

*#3 Search ((review[Title/Abstract]) OR systematic review[Title/Abstract]) OR meta[Title/Abstract].*

*#4 #1 AND #2 AND #3.*

### Type of study

We included systematic reviews with or without meta-analysis.

### Participants

Patients with a confirmed diagnosis of UC, regardless of disease duration and severity, were included.

### Interventions

We included SRs where TCM interventions were used, including Chinese herbal medicine, extracts from herb mixtures, individual herbs, Chinese patent medicine, or herb compounds prescribed by Chinese medicine practitioners, regardless of the potential mechanisms of action. Eligible treatments allowed the intervention to be combined with conventional medicine or placebo. No limitation on drug dosage form and route of administration.

### Outcome measures

Systematic reviews were considered where outcome measures included clinical effectiveness, TCM syndromes, inflammatory levels, immunological indicators, blood indicators, electronic endoscopy results, intestinal flora, clinical symptom and incidence of adverse reactions.

### Exclusion criteria

Studies that involved comorbidity, in particular intestinal diseases, such as CD, schistosomiasis, bacillary dysentery, and intestinal tuberculosis, were excluded. Interventions involving herbal medicines not prescribed according to TCM theory or by Chinese medicine practitioners, were also excluded.

### Data extraction and methodological quality

Microsoft Excel was used for data extraction and bubble plot creation. Two researchers independently screened the studies, extracted the data, and evaluated the report. A third author was consulted if there were discrepancies. Relevant data extracted included author(s), year of publication, country of origin (based on primary author’s affiliation), total number of patients, diagnostic criteria, severity of illness, medication in intervention group and control group, outcomes, safety evaluation, quality assessment tools, and funding support. We used AMSTAR 2 [15] (a measurement tool to assess systematic reviews 2) as a quality assessment tool, and two experts assessed each study. If there were any discrepancies between the two reviewers, a third reviewer was consulted.

AMSTAR 2 was used to critically appraise the methodological quality of the SRs. AMSTAR 2 contains 16 items that appraise critical flow and bias using ratings of “yes”, “partial yes” or “no”. Using this rating system, the overall confidence for the SRs were assessed as “high” (no or non-critical weakness in all items), “moderate” (more than one non-critical weakness among all the items), “low” (one critical flaw with or without non-critical weakness), or “critically low” (more than one critical flaw with or without non-critical weakness) [15].

### Graphical visualization of results

Findings for included SRs were summarized in:

(a) tables describing the characteristics and outcomes of the included SRs;

(b) graphical display of the results of literature quality evaluation, with mapping based on bubble plots. Display information includes: (1) effectiveness trend as ‘no difference’, ‘potentially effective’, and ‘effective’ in x-axis. (2) estimated size of the literature y-axis; and (3) the bubble size as per AMSTAR 2 assessment, representing Chinese herbal therapy intervention type.

## Results

### Overall assessment of included systematic reviews

**Fig. 1:**
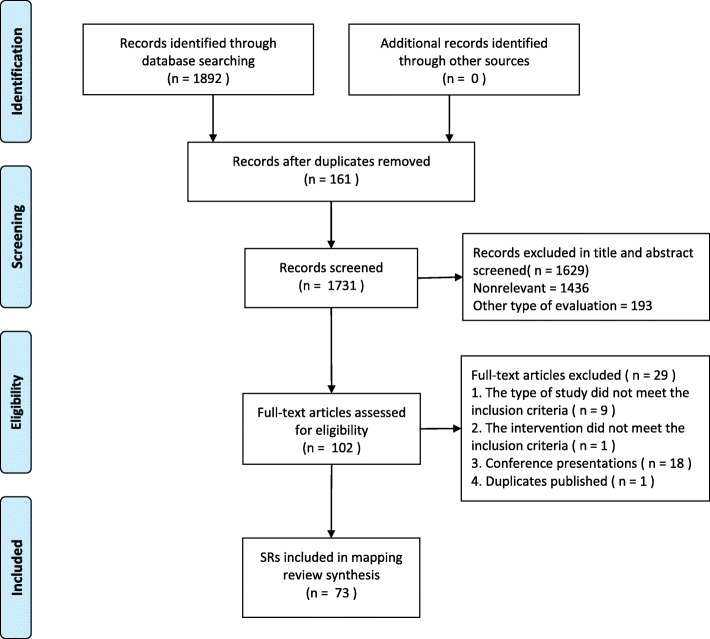
Flow diagram of literature searches

Our search identified 1892 potential systematic reviews (Fig. 1). After removing duplicate publications, the titles and abstracts of 1731 reviews were screened for relevancy. Of these, the full-text articles of 102 reviews were reviewed for eligibility. Finally, 73 SRs were included in our final review (Fig. 1) [16–88]. The basic characteristics of the included SRs are shown in Table 2 in Appendix 1. Among the included studies, one systematic review reported participants in the active phase of UC [16], one reported participants in remission [17], whilst another reported participants that were in a non-acute occurrence of UC [18]. 10 SRs did not limit the severity of the disease [19–28], whilst other reviews did not report severity of UC. In most studies, the control group included the first-line medication of conventional medicine, such as mesalamines, amino preparations, hormone preparations, and probiotics, whilst some studies used other herbal medicines and placebo. The administration routes of the intervention groups included oral administration, enema, embolization, injection, ultrasound induction and acupoint application.

### Literature publication

**Fig. 2:**
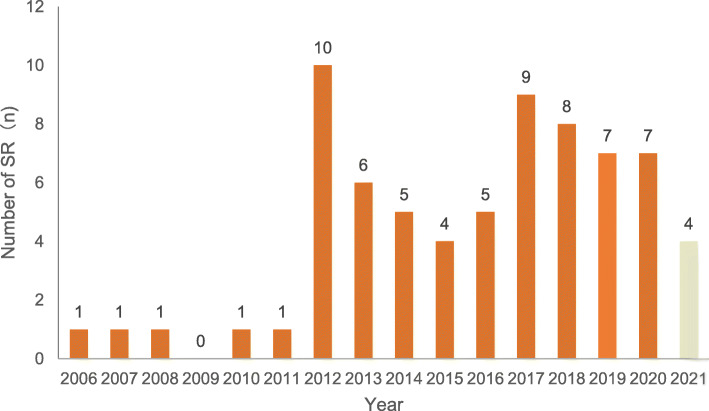
Systematic reviews of TCM in the treatment of UC published to 2021

Figure 2 shows the number of included SRs of TCM in the treatment of UC by published year. Most of the 73 included SRs were published in the last 8 years, with the majority in 2012. The number of published SRs each year has increased from 0 to 1 from 2006 to 2011. Interestingly, the number of SRs published each year has significantly increased over the last decade. Results for 2021 are not complete, and reflect SRs published up to 16 March 2021.

### Quality of included systematic reviews

**Fig. 3:**
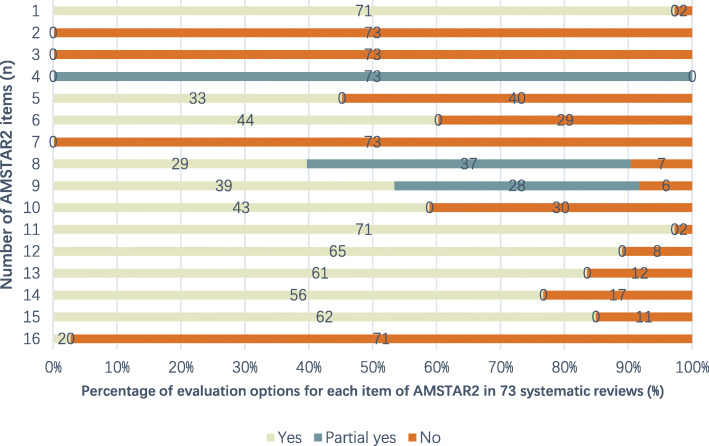
Methodological quality of systematic reviews with meta-analysis of TCM in the treatment of UC Measured with AMSTAR 2 tool

AMSTAR 2 was used to critically appraise the reporting quality of each included systematic review and all reviews were found to be of critically low quality (Fig. 3). Whilst majority of reviews assessed the risk of bias in interpreting results and reported any conflicts of interest, we found that no reviews mentioned study lists and reported exclusion criteria in the review methods.

### Outcomes

Outcome indicators (listed in Table 3 in Appendix 2) included clinical effectiveness, incidence of adverse reactions, TCM syndromes score, levels of inflammatory factors, immunological indicators, blood indicators, results of electronic endoscopy, intestinal flora, and description of clinical symptoms. A total of 44 outcomes were identified as having inconsistent reporting and measurements. Continuous variables were presented as mean with/without standard deviation, and dichotomous variables were described as a number or percentage. We summarized all the reported outcomes and presented the results with descriptive statistics (Table 1). Commonly reported outcomes included clinical effects (71/73, 97.3%), adverse reaction rate (42/73, 57.5%), clinical symptoms (19/73, 26.0%), serum inflammatory cytokines levels (9/73, 12.3%), infection screening (8/73,11.0), performance of colonoscopy (24/73, 32.9%), immune factor level (4/73, 5.5%), blood routine (4/73, 5.5%), and level of intestinal flora (1/73, 1.4%).

**Table 1:** Outcomes reported in 73 reviews evaluating TCM in the treatment of UC

		Outcomes	Number of reviews (%)
Clinical effects	1	Clinical effectiveness	28 (38.4)
	2	Total effectiveness	48 (65.8)
	3	Cure rate	12 (16.4)
	4	Significant effectiveness	2 (2.7)
	5	Inefficiency rate	2 (2.7)
	6	Recurrence rate	23 (31.5)
Adverse reaction	7	Adverse reaction rate	42 (57.5)
Immune factor levels	8	TNF-a	5 (6.8)
	9	IL-6	5 (6.8)
	10	IL-8	3 (4.1)
	11	IL-10	1 (1.4)
	12	IL-13	1 (1.4)
	13	IL-17	3 (4.1)
	14	IL-23	3 (4.1)
Infection screening	15	CRP	7 (9.6)
	16	ESR	5 (6.8)
Performance of colonoscopy	17	Performance of colonoscopy	20 (27.4)
	18	Mucosal biopsy score	4 (5.5)
	19	Efficacy of mucosal lesions	1 (1.4)
Clinical symptoms	20	TCM syndrome score	14 (19.2)
	21	DAI	9 (12.3)
	22	Abdominal pain	6 (8.2)
	23	Diarrhea	6 (8.2)
	24	Pus and blood stool	6 (8.2)
	25	Tenesmus	3 (4.1)
	26	Time of bellyache disappearance	3 (4.1)
	27	Time of diarrhea disappearance	3 (4.1)
	28	Time of hematochezia disappearance	2 (2.7)
	29	Time of fever disappearance	2 (2.7)
	30	Defecate occult blood	2 (2.7)
	31	Total symptom score before and after treatment	1 (1.4)
	32	Geboes index	1 (1.4)
	33	Symptom relief time	1 (1.4)
	34	Intervention treatment	1 (1.4)
Serum inflammatory cytokines levels	35	IgG	4 (5.5)
	36	IgA	3 (4.1)
	37	IgM	2 (2.7)
Blood routine	38	Negative conversion rate of White blood cells and red blood cells in stool routine	1 (1.4)
	39	Whole blood viscosity score	1 (1.4)
	40	Plasma viscosity	1 (1.4)
Level of intestinal flora	41	Bifidobacterium level	1 (1.4)
	42	Lactobacillus level	1 (1.4)
	43	Enterococcus level	1 (1.4)
	44	*E. coli* level	1 (1.4)

Abbreviations: *DAI* DNA-dependent activator of IFN-regulatory factors; *IL* inflammatory factors levels of interleukin; *ESR* erythrocyte sedimentation rate; *CRP* C-reaction protein; *IgA* immunoglobulin A; *IgM* immunoglobulin M; *IgG* immunoglobulin G

Outcome indicators were further classified. Clinical effects included clinical effectiveness, total effectiveness, cure rate, significant effectiveness, inefficiency rate and recurrence rate. Clinical symptoms included details such as TCM syndrome, total symptom score before and after treatment, disappearance of mucopurulent bloody stool/abdominal pain/diarrhea/tenesmus, DAI and Geboes index. Serum inflammatory cytokines levels included IgA/M/G. Immune factor levels included TNF-a and IL-6/8/10/13/17/23. Infection screening included ESR and CRP. Compared with conventional medicine, 68 SRs reported positive outcomes. Two SRs reported no difference in clinical efficacy compared with conventional medicine [29, 30]. One review reported no difference in ESR compared with conventional medicine [31]. Intervention measures in 34 SRs included only TCM treatment, whilst the remaining integrated both traditional Chinese and conventional medicine in treatment. 37 SRs reported safety outcomes of which the majority were positive, and 9 reported a negative incidence of adverse reaction rate [19, 20, 24, 25, 30, 32–35]. Safety outcomes were not reported in 25 SRs [22, 23, 28, 36–57]. Publication bias was not investigated in 11 reviews [18, 22, 29, 30, 41, 47, 54, 58–61]. There were 30 SRs that did not report funding support [16, 17, 19, 22, 24, 27, 36–38, 40, 41, 43–46, 48, 49, 54–56, 59, 62–70], and 14 of them were dissertations [16, 17, 22, 37, 40, 45, 49, 60, 62, 63, 65, 68–70].

### Evidence Mapping

**Fig. 4:**
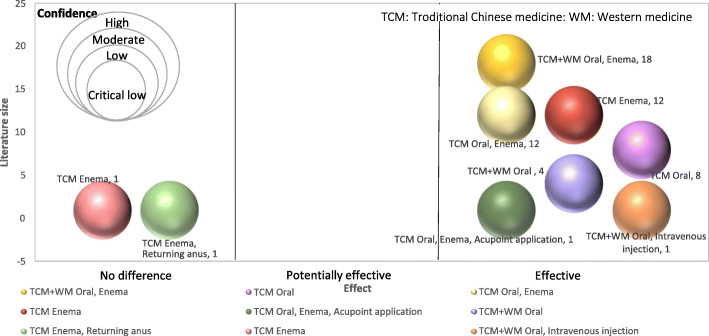
Evidence map of TCM in the treatment of UC. The bubble plot in Fig. 4 summarizes TCM treatment for UC in systematic reviews published as of 2021, the estimated size of the literature (y-axis), the effectiveness trend according to reviews (x-axis), and the confidence of systematic reviews (bubble size)

Evidence mapping focused on clinical effectiveness outcomes. We evaluated the effectiveness, literature size and confidence level for each intervention identified in the SRs. The most common treatment in randomized controlled trials in the SRs were oral administration and enema interventions with Chinese and conventional medicine (*n* = 18) [19–21, 27, 34, 40, 46, 53, 66–68, 70–76]. 12 studies involved TCM retention enema treatments only (*n* = 12) [30, 32, 41–44, 48, 55, 64, 77–79] and another 12 studies included both oral administration and enema with TCM (n = 12) [17, 18, 28, 31, 57, 62, 63, 65, 69, 80–82]. 16 reviews did not report specific routes of administration in the intervention group [22–24, 38, 47, 50–52, 54, 58–60, 83–86]. The use of TCM treatment in UC is positive but the quality of SRs are low. The evidence mapping showed that there is limited number of studies using between TCM and conventional medicine combined with multiple routes of administration is inconclusive, indicating a need for more original research in this area (Fig. 4).

## Discussion

There is a constant demand for health care when it comes to chronic disease. Patients with UC tend to have long term effects with increased risk of cancer, which may develop into UC-related colorectal cancer (UC-CRC). UC-CRC is one of the most serious complications in patients with long-term UC [89]. Among all cancers, colorectal cancer is a high-cost, high-burden malignancy that takes a heavy toll on health care systems and patients [90–92]. Evidence mapping of SRs in TCM is therefore critical to understand where further research should be focused to ensure the financial and health toll on patients with UC.

### Main findings

In accessing the clinical effectiveness of treatment options for UC in the published SRs, we found that a majority of clinical trials used TCM as intervention through a variety of routes of administrations. Our evidence mapping showed that oral administration and enema with both Chinese and conventional medicine was most widely studied (*n* = 18). Oral combined enema was the most widely used route of administration in the trials. TCM only was the most common intervention (*n* = 34), followed by the combination of TCM and conventional medicine (*n* = 23). The overall confidence level for each review was limited. So whilst TCM treatments may be effective in UC, more research is needed to determine whether it can be recommended to patients.

The conclusion of this evidence mapping review however cannot provide recommendations for clinical practice due to insufficient strength of evidence and limitation of research type. AMSTAR2 was only used to evaluate the quality of methodological reports and not the efficacy of medicine. A mapping review can help in describing the research field and provide a basis for an informed decision about whether to undertake an in-depth review and synthesis of all or a subset of studies. However, its analysis only characterizes quantity and quality of literature rather than offering recommendations for practice and future research though the quantitative synthesis. The SR can only answer one specific PICOS clinical question at a time. We chose to include SRs as the study type in our evidence mapping as it can provide a broad and often comprehensive summation of a topic area, providing value for those coming to a subject for the first time. There are some shortcomings attributed to mapping reviews, specifically characterizing at a broad descriptive level. This can oversimplify the picture or mask considerable variation (heterogeneity) between studies and their findings, depending on the degree of specificity of the coding process [13]. The purpose of the statistics of outcomes is to sort out the outcomes and degree of concern in the past clinical trials of TCM in the treatment of UC: the greater the percentage, the more attention it is likely to receive. It provides evidence for the establishment of ‘core outcome set (COS)’, that is a set of minimum and consensus standardized indicators that should be measured and reported by all clinical trials in a specific health or healthcare field. However, we should consider that the choice of outcomes in clinical trials depends on the stage of UC, active or in remission, and the selection of primary and secondary outcomes.

It is noteworthy that the number of SRs on TCM in the treatment of UC has increased since 2012. Prior to this, the number of trials in 2006–2011 was 0–1. Previously UC had a higher incidence in Western countries, but there has been a dramatic increase in the incident rate of UC in Asia in recent years. Because the disease is difficult to cure, easy to relapse, and the risk of cancer lesions is high, it often takes lifelong medication. There are major concerns in diagnosis and treatment of UC worldwide, with research currently being a hot topic in the field of gastroenterology.

Various guidelines for the diagnosis and treatment of UC are constantly being revised. In 2004, the American Gastroenterology Association (AGA) revised “Ulcerative colitis practice guidelines in adults” [93], British Society of Gastroenterology (BSG) reviewed “Guidelines for the management of inflammatory bowel disease in adults” [94], and 2010 World Gastroenterology Organization Practice Guidelines for the Diagnosis and Management of IBD [95]. In 2007, Chinese Society of Gastroenterology (CSGE) “the Consensus Opinions on the Diagnostic and Treatment Specifications for Inflammatory Bowel Disease in China” [96]. In 2010, after repeated discussions and practice, the “Consensus on the Diagnosis and Treatment of Integrated and conventional Medicine for Ulcerative Colitis” [97] was formed. This is the first formal consensus opinion on the treatment of UC in Chinese medicine. This may be one of the reasons why the Chinese medicine field began to pay attention to the disease, and the number of SRs increased from 2012.

### Strength and limitations

#### Strength

The systematic evaluation of relevant topics was searched. We used the PICOS format to organize the available information and describe the results applied to more specific scenarios according to current clinical practice. Our study included 73 SRs, and it is difficult to describe the information involved by general methods. In fields where there are enormous amounts of available information, the bubble plots are a good option. Mapping methodologies describe the quality of included SRs, and we could combine the results of all conducted studies in the field. We assessed the quality of included reviews with AMSTAR 2. This approach allowed results to be displayed on a bubble plot for each systematic review with respect to the other ones with the same comparison, providing a quick view of the existing evidence and their quality.

#### Limitations

There were several limitations to this research. Firstly, the results of our review need to be interpreted carefully because of the many characteristics in the SRs. Current evidence for interventions used in UC therefore cannot be considered conclusive and show a clear need for further research. Second, our study has some methodologic limitations. When drawing the evidence map, we included domains of estimated overall clinical effectiveness (x-axis), literature size (y-axis), and the confidence of the evidence (size of the bubble). When determining clinical effectiveness, we relied on the results of 58 SRs that potentially included biased information. The clinical effectiveness depends on these outcome indicators, including clinical effectiveness, total effectiveness, cure rate, significant effectiveness, inefficiency rate. Moreover, the AMSTAR 2, used to determine the confidence level, was originally developed to assess the appropriateness of reporting SRs and not to evaluate the confidence level of SRs. We acknowledge that there may be more efficient ways of drawing an evidence map.

Since only a few SRs in the included studies showed the stage of the disease, we did not evaluate characteristics on subgroups in acute and non-acute phase of UC. The main reason may be that many RCTs did not clearly define the disease stages of participants, which is a methodological demerit in this field. We hope that more RCTs with definite inclusion and exclusion criteria can be carried out to evaluate the effects on subgroups in the future.

## Conclusion

In conclusion, oral administration, in combination with enema in both Chinese and conventional medicine, has been the most frequently tested intervention in TCM for UC. But on the basis of current evidence, this therapy can only be recommended cautiously. The low grade methodology quality of the included SRs in our mapping research cannot provide a high level of evidence to recommend in clinical practice. The major issue was that SRs failed to report the location of the disease, the type of the disease, and the route of administration of the intervention. Outcome indicators were also not uniformly reported, and the exact effect of Chinese medicine on UC cannot be derived from available evidence. Further evaluation of the effects of TCM is needed, either alone or in combination with conventional medicine, or via multiple administration routes. Low quality RCTs of TCM in the treatment of UC is of concern. As no valid conclusion can be drawn, it is a waste of energy of authors. Clinicians should carefully execute the trial and report details of the research process. They should also be aware that negative published data do not change the reputation of these authors, whilst do not annoying editors and readers. Before the systematic evaluation, the researchers should register the protocol and list the excluded literatures. In the future, we believe when high quality clinical studies are conducted of various Chinese herbal interventions, superior evidence will be available to confirm the results of these trials. In the meanwhile, evidence mapping is a useful and reliable methodology to identify and present the current evidence about therapeutic interventions. The results can help us accurately locate the focal point and insufficiency of current research in the field.

### Supplementary Information

**Additional file 1.**

## Data Availability

All data generated or analyzed during this study are included in this published article and its supplementary information files.

## Appendix 1

**Table 2:** Summary of the included systematic reviews

No.	Study ID (first author, year)	Interventions	Review objectives (quote from the original paper)	Population	Intervention (TCM used)	Comparator	Number of studies
1	Gong Y2014	Decoction	To evaluate the efficacy and safety of Sishen Pill in the treatment of UC.	*n* = 839	Sishen Pills	SASP, Gubenyichang Tablets, Bupiyichang Pill, Hydrocortisone	10 RCTs
2	Li WH2013	Decoction	To evaluate the efficacy and safety of Pulsatilla Decoction for UC.	*n* = 1124	Pulsatilla Decoction	SASP	10 RCTs
3	Zuo HB2013	Decoction	To evaluate the efficacy and safety of Pulsatilla Decoction for UC.	*n* = 1480	Pulsatilla Decoction	SASP, 5-ASA, Hormone preparation	12 RCTs
4	Pei QW2012	Decoction	To evaluate the effectiveness and safety of Jiajian Banxia Xiexin decoction forUC.	*n* = 736	Banxia Xiexin Decoction, Danggui Shaoyao Powder, Taohong Siwu Decoction	SASP, Bifico, Hormone preparation, Mesalazine	8 RCTs
5	Wen Y2017	Decoction, Chinese patent medicine	To make a Meta-analysis of the effectiveness and safety of Shenling Baizhu Powder for the treatment of UC, thus to provide evidence for the clinical treatment of UC.	*n* = 1498	Shenling Baizhu Powder	SASP, Mesalazine, Hormone preparation, Osalazine	19 RCTs
6	Chen K2016	Chinese patent medicine	Generally evaluate the therapeutic effect of Shenling Baizhu Powder combined with Western Medcine on UC.	*n* = 884	Shenling Baizhu Powder	SASP, Mesalazine	10 RCTs
7	Lu2017	Chinese patent medicine	To evaluate the clinical efficacy and safety of Shenling Baizhu powder in the treatment of UC by means of evidence-based medicine.	*n* = 1736	Shenling Baizhu Powder	Mezalazine, SASP, Metronidazole, Osalazina	20 RCTs
8	Wang XY2017	Chinese patent medicine	To evaluate the clinical efficacy and safety of Shenling Baizhu Powder in the treatment of UC.	*n* = 1041	Shenling Baizhu Powder	SASP, Mesalazine	12 RCTs
9	Wei Y2018	Decoction, Chinese patent medicine	To evaluate the clinical efficacy of retention enema with TCM in the treatment of UC.	*n* = 1447	Baishao Qiwu Granule, Changyankang I, Compound Xuejie Enema on the Blood Rheology, Colon soup, Kuijie enema, Lianbei Mixture, Qingjie Qushi Decoction, Shibai Shenbai Decoction	SASP, Hormones, gentamicin, dexamethasone, furazolidone, montmorillonite powder,smecta	16 RCTs
10	Zhang LH2018	Decoction, Chinese patent medicine	To evaluate Fuzilizhong decoction and its modified decoction systematically and summarize the clinical efficacy and safety of the treatment of UC.	*n* = 688	Fuzi Lizhong decoction, Sishen Pill, Kangfuxin solution	SASP, Kangfuxin	8 RCTs
11	Li HB2017	Chinese patent medicine	To systematically evaluate the clinical efficacy and safety of Compound Huangbo liquid combined with chemical medicine in the treatment of UC, and to provide evidence-based reference	*n* = 737	Compound Huangbo liquid, Mezalazine	SASP, Mezalazine, 5-ASA, Hormones, immunosuppressants, physiological saline	8 RCTs
12	Li HB2018	Chinese patent medicine	To systematically evaluate therapeutic efficacy and safety of Compound kushen colon-release capsule versus related chemical drugs in treatment of UC.	*n* = 649	Compound kushen colon-release capsule	SASP, Mezalazine, placebo	9 RCTs
13	Huang FMZ2014	Decoction	To evaluate the efficacy of JieDuXiaoYongFa and the variation of relevant indicators compared to conventional medicine for the treatment of UC.	*n* = 1884	NR	SASP, Mezalazine, Hormones, Gentamicin, Penicillin, Smecta	23 RCTs
14	Li HB-a2018	Chinese patent medicine	To systematically evalutate the clinical efficacy of Kangfuxin Liquid versus aminosalicylic acid in treating UC, in order to provide scientific basis for clinical promotion.	*n* = 806	Kangfuxin Liquid	SASP, 5-ASA, Folic acid, Metronidazole	9 RCTs
15	Liu G2011	Chinese patent medicine	To evaluate the effect of the treatment based on Kangfuxin liquid in UC.	*n* = 607	Kangfuxin Liquid	NR	8 RCTs
16	Gu SZ2018	Decoction, Chinese patent medicine	To systematically evaluate the efficacy and safety of the RCT of oral Chinese medicine for the treatment of UC.	*n* = 1703	Kuijie Decoction, Changyankang, Jianpi Yuchang Decoction, Qingchang Huashi Decoction, Sijunzi Decoction, Xianglian Zhixie Tablet, Wenjing Decoction, Juyuan Decoction, Wumei Pill, Qingre Lishi Recipe, Baiji Yukui Decoction, Changqingshu Decoction, Jianpi Huazhi Pill, Kuijiening, Zhenren Yangzang Decoction, Wenshen Jianpi Decoction, Wenyang Yuyang Decoction, Fuzheng Quxie Decoction.	SASP, Mezalazine	20 RCTs
17	Xiong AQ2011	Decoction	Objective evaluation of the treatment of UC with TCM.	*n* = 582	Wumei Baijiang Decoction, Shaoyao Decoction, Jianpi Decoction, Qingchang Yuyang Decoction, Qingchang Liangxue Decoction, Sijunzi Decoction, Tongxieyao Decoction, Jianpi Qushi Huazhuo Decoction.	Mezalazine	8 RCTs
18	Lv C2014	NR	To evaluate the efficacy and safety of clearing away intestinal dampness and heat methods on UC.	*n* = 1797	NR	SASP, Mezalazine, Metronidazole, Osalazina	20 RCTs
19	Wang DY2011	Decoction, Chinese patent medicine	To evaluate heat spleen with wet method in active treatment the curative effect of UC and security, to reveal the advantages of the method in active treatment of UC activity for TCM treatment of UC to provide the basis of evidence-based medicine.	*n* = 2641	Anchang Zhixie Decoction, Shenling Baizhu Decoction, Changyuning Granule, Gegen Qinlian Decoction, Kuijie Decoction, Lipi Yuyang Decoction, Baitouweng Decoction, Mankuining, Qinghua Changyin	SASP, Mezalazine, Hormones, Smecta,Metronidazole, Gentamicin, Placebo	36 RCTs
20	Liu TW2016	Decoction	To explore the effectiveness and safety of Paeoniae decoction in the treatment of UC.	*n* = 1181	Shaoyao Decoction	BaWei XiLei Powder, SASP, Metronidazole, berberine, hydrocortisone, oxalazine, hormones	17 RCTs
21	Yang L2017	Decoction	To evaluate the efficacy of Shaoyao Decoction in the therapy of UC.	*n* = 637	Shaoyao Decoction	SASP, Mezalazine	9 RCTs
22	Zhang WN2017	Decoction, Oitment	To evaluate the efficacy of Tongxie Yaofang in the therapy of UC.	*n* = 696	prescription for treating diarrhoea with abdominal pain, Baizhu Shaoyao Powder, Changyanling	SASP, Probiotics, Norfloxacin, Vitamin, Smecta	8 RCTs
23	Chen F2012	Decoction	To evaluate the kidney and spleen method in the treatment of remission of UC efficacy and safety.	*n* = 2928	Wenyang Yiqi Jiedu Decoction, Wumei Pill, Zhenpi Decoction, Huangqi Jianzhong Decoction, Bupi Yichang Decoction, Wenshen Hezhong Decoction, Fuzi Lizhong Decoction, Sishen Pill, Shenling Baizhu Powder, Buzhong Yiqi Decoction, Lizhong Decoction, Kuijieling, Xileisan, Qiwei Baizhu Powder, Jianpi Yishen Formula, Jiechang Kang, Wenbu Zhixie Decoction, Weichangning Decoction, Huangtu Decoction, Jianpi Lichang Decoction, Tiaozhong Lichang Decoction, Jiedu Shengji Decoction, Lianli Decoction and Zhenren Yangzang Decoction	SASP, Mezalazine, Xileisan, Smecta, Norfloxacin, Hormone, Gentamicin	35 RCTs
24	Yan SG2013	Decoction	To evaluate the efficacy of Wumei Pill in the therapy of UC.	*n* = 1170	Wumei Pill	SASP, Bupi Yichang Pill, Amoxicillin	10 RCTs
25	Xiong J2008	Decoction	To evaluate the efficacy of Wumei Pill in the therapy of UC.	*n* = 1159	SASP, Wumei Pill, Zhenren Yangzang Decoction, Qiwei Baizhu, Liujunzi Decoction, Sishen Pill	SASP, Hormone	10 RCTs
26	Chen MY2018	Chinese patent medicine	To systematically evaluate the clinical efficacy and safety of Xilei Powder compared with mesalamine in the treatment of UC.	*n* = 373	Xilei Powder	Mezalazine	6 RCTs
27	Chen MY2018-a	Chinese patent medicine	Meta-analysis of Xileisan combined with Mesalazine in the treatment of UC.	*n* = 840	Xilei Powder	SASP, Hormone	12 RCTs
28	Cui DJ2012	Chinese patent medicine	To evaluate the efficacy and safety of Xilei powder on UC.	n = 83	Xilei Powder	Mezalazine	2 RCTs
29	Ma XM2012	Chinese patent medicine	To systematically evaluate the clinical efficacy of Xileisan in the treatment of UC.	*n* = 1476	Xilei Powder	SASP, 5-ASA, Smecta, Hormone, Antibiotic	21 RCTs
30	Lai YL2013	Decoction	To evaluate the clinical efficacy of TCM with Xinkai Kujiang method in the treatment of UC.	*n* = 551	Wumei Pill, Lianli Wumei Decoction, Chaigui Ganjiang Decoction	SASP, Mezalazine	9 RCTs
31	Zhu JB2016	Decoction	To make a systematic review on the clinical effect and safety of modifiedBanxia Xiexin Decoction in the treatment of UC.	*n* = 1200	Banxia Xiexin Decoction	NR	14 RCTs
32	Huang ZB2014	Chinese patent medicine	To systematically evaluate the effectiveness of Yunnan Baiyao in treating UC.	*n* = 1463	Yunnan white Drug	SASP, 5-ASA	20 RCTs
33	Qi J2016	Decoction	To evaluate the clinical efficacy of Zhenren Yangzang Decoction in the treatment of UC.	*n* = 209	Zhenren Yangzang Decoction	SASP	3 RCTs
34	luo Y2012	NR	Evaluation the efficicy and safety about the Chinese and Western method of treatment on UC.	*n* = 7740	NR	NR	113 RCTs
35	Xu P2015	Decoction, Chinese patent medicine	1 To sum up the results of the RCT about the treatments of UC with integrative medicine.2 Using Mete-analysis methods to evaluate the effectiveness,safety and the rates of recurrence,in order to provide reference and guidance for clinical treatment of UC.	*n* = 1696	Pulsatilla Decoction, Kangfuxin Liquid, Hongteng Decoction, Puqin Baijiang Decoction	conventional medicine	20 RCTs
36	Gong YD2012	NR	Evaluate wether conventional medicine combined TCM therapy more advantageous than simple Western medicine therapy.	*n* = 1897	NR	conventional medicine	16 RCTs
37	Ma DZ2015	NR	Comprehensive evaluation of the clinical efficacy of retention enema with TCM in the adjuvant treatment of UC.	*n* = 1358	NR	conventional medicine	18 RCTs
38	Huang SG2010	Decoction, Chinese patent medicine	Applying the method of Meta-Analysis, generally evaluation the therapeutic effect of retention-enema of Chinese herb treating UC.	*n* = 875	Danshen enema, Hongteng mixture, Kuju solution, Kuijieqing enema, Huangqi Decoction, Baitouweng Decoction	SASP, Hormone, Antibiotic, Sulfamethoxazole	7 RCTs
39	Ni XX2019	Decoction, Chinese patent medicine	To systematically evaluate the efficacy and safety of retention enema with Chinese materia in the treatment of UC.	*n* = 3110	Yunnan white Drug, Xilei Powder, Qibei Mixture, Zhikang Capsule, Shenling Baizhu Powder	conventional medicine	36 RCTs
40	Jiang T2006	NR	To assess the theraoeutic effectiveness of retention enema with TCM in the treatment of UC.	*n* = 2092	NR	SASP, Hormone, Antibiotic	23 RCTs
41	Zhu XG2012	Decoction, Chinese patent medicine	Comprehensive evaluation of clinical efficacy of retention enema with TCM in the treatment of UC.	*n* = 1584	Yunnan White Drug, Jiechang Decoction, Kuijiekang, Kuju Liquid, Yuchang Zhengchang Decoction, Tongguan Decoction, Yasanzi Sanhuang Decoction, Hongteng Mixture, Yuyang Anchang Decoction, Danshen enema Liquid, Xilei Liquid, Kuijieqing enema Liquid, Pulsatilla Decoction, Huangqi Decoction.	SASP, Hormone, Antibiotic, Smecta, Sulfamethoxazole compound	15 RCTs
42	You WF2017	NR	Quantitative analysis of clinical efficacy of retention enema of TCM for UC based on evidence-based medicine methodology.	*n* = 988	NR	NR	7 RCTs
43	Cui DJ-a2012	Chinese patent medicine	To evaluate the efficacy and safety of Bupi Yichang Pill in the therapy of UC.	*n* = 596	Bupi Yichang Pill	SASP, Mezalazine, balsalazide	6 RCTs
44	WuZl2017	Decoction, Chinese patent medicine	To evaluate the therapeutic effect of TCM enema combined with mesalazine in treating UC.	*n* = 1521	Changkui Decoction, Qingchang Huashi Decoction, Qingchang Yuyang Decoction, Shenling Baizhu Powder and Baitouweng Decoction	Mezalazine	22 RCTs
45	Huang HJ2012	NR	The objective of this study was to systematicly ewduate the clinical therapeutic effcct of Chinese materia medica and western drugs used in retention enema for treating UC.	*n* = 476	NR	NR	5 RCTs
46	Zha AS2015	NR	The aim of this study was to evaluate the safety and efficacy of Huoxue Huayumethod of TCM in the treatment of UC.	n = 1897	NR	Mezalazine, SASP, Hormones, Antibiotics, Metronidazole	20 RCTs
47	Hou LW2017	Decoction	To review systematically the therapeutic effects and safety on UC treated with the oral administration and enema with TCM	*n* = 1507	Tiaoqi Jiedu Decoction, Yuchang Decoction, Shenling Baizhu Powder, Gegen Qinlian Decoction, Shaoyao Decoction, Changyan Decoction, Jianpi Lichang Decoction, Self-made Xiaoulcer, Gegen Qinlian Wutan Decoction	SASP, Hormones, Ampicillin	18 RCTs
48	Hou LW2015	Decoction, Chinese patent medicine	To systematically evaluate clinical efficacy of oral medicine decoction with enema treatment of UC.	*n* = 856	Colon Ning Mixture, Yuyangning Decoction, Tongxie Yaofang, Shenling Baizhu Powder, Shaoyao Decoction, Gegen Qinlian Decoction, Gegen Qinlian Wutan Decoction	SASP, Hormones, Antibiotic	11 RCTs
49	Gan YK2015	Decoction	To evaluate the efficacy of TCM for oral compared with Mesalazine in thetreatment of UC through meta-analysis.	*n* = 543	Changqingshu Decoction, Sijunzi Decoction and Tongxie Decoction, Qingchang Yuyang Decoction, Wumei Baicai Decoction, Jianpi Decoction, Qingchang Liangxue Decoction, Jianpi Qushihuo Decoction	Mezalazine	7 RCTs
50	Zhu JM2011	NR	To evaluate the clinical efficacy of Chinese medicine treatment on UC.	*n* = 2702	NR	SASP, Basalazide, Mesalazine, Smecta, Antibiotics, Hormones, Vitamins	33 RCTs
51	Wang DY2013	Decoction, Chinese patent medicine	To evaluate the curative effect and safety of TCM Heat-Clearing and Damp-Excreting and Spleen-Strengthening Method for the treatment of active UC.	*n* = 896	Modified Yuyang Decoction, Huangqi Jianzhong Decoction, Huoxue Lichang Decoction, Jianpi Lishi Decoction, Kuijieling No. 1, Qibaiyichang Decoction, Qini Yuyang Decoction, Baitouweng Decoction, Kuijiefukang Decoction, Qingre Lishi Yichang Decoction, Baitouweng Decoction, Liuhe Decoction, Jianpiyukui Decoction, Chinese Herbal Enema Prescription	SASP, Smecta, Hormone, Ciprofloxacin	13 RCTs
52	Wang Y2018	NR	To evaluate the regulation of gut flora in patients with UC on TCM.	*n* = 392	NR	SASP, Mezalazine	5 RCTs
53	He M2007	NR	Comparing the clinical efficacy of TCM preparation and SASP in the treatment of UC.	*n* = 611	NR	SASP	7 RCTs
54	Pei QW2013	Decoction, Chinese patent medicine	To evaluate the effectiveness and safety of method of chinese herbs oral therapy for treating UC.	*n* = 1923	Tongxiening Granule, Fuling Powder, Wumei Pill, Mahuang Fuzi Xixin Decoction, Compound Kushen Colon-dissolving Capsule, Xuefu Zhuyu Decoction, Shaoyao Decoction, Kuijie Recurrent Decoction, Ulcer Powder, Zhuche Pill, Guipi Decoction, Changpikang, Qixian Anchang Decoction, Jianpi Zaoshi Decoction, Kuijietong Decoction, QinGeng Chunpi Decoction Liquor, Coix Root and Fructus Aconiti Patriniae Powder, Changyankang Oral Liquid, Warming Spleen Decoction, Banxia Xiexin Decoction, Danggui Shaoyao Powder, Taohong Siwu Decoction, Qiwei Baizhu Powder	SASP, Hormones, Oxalazine, Folic acid, miya, Mesalazine	23 RCTs
55	Yang AX2006	NR	To compare clinical therapeutic effects of simple TCM and simple conventional medicines on UC.	*n* = 1237	NR	NR	11 RCTs
56	Zhu L2012	NR	To compare clinical therapeutic effects of simple TCM and 5-ASA on UC.	*n* = 739	NR	NR	11 RCTs
57	Jia JW2019	Decoction, Chinese patent medicine	Systematic evaluation of the clinical efficacy of TCM retention enema in the treatment of UC.	*n* = 1450	Colon Qingfang, Kuijie Enema Decoction, Qingre Jiedu Decoction, Ulcer Powder, Sanqi Zicao Decoction, Diyu Charcoal with Baiji, Sanhuang Decoction, Kuijie Decoction, Baishao Licorice Decoction	SASP, Metronidazole, Dexamethasone, Hydrocortisone, Mesalazine, Gentamicin, Tinidazole, Gentamicin sulfate, Oxalazine	16 RCTs
58	Li L2019	Decoction	Systematic evaluation of the efficacy and safety of TCM for clearing away heat and dampness combined with conventional medicine in the treatment of UC.	*n* = 1176	Bai Tou Weng Decoction, Qingluo Huachang Decoction, Gegen Qinlian Decoction, Yiqi Qingchang Decoction, Baitouweng plus Gancao and Ejiao Decoction, Shaoyao Decoction, Qingchang Powder, Qingchi Powder, Qingre Changyu Decoction, Yu Chang Ning capsule, Kuiyu Decoction	SASP, Mezalazine	15 RCTs
59	Chen MJ2019	Decoction, Chinese patent medicine	To systematically evaluate clinical effects of proprietary Chinese medicine containing *Sophora Flavescens* on UC.	*n* = 883	Composite Sophora Colon-soluble Capsules, Kuh-seng Injection, Kuh-seng enema, Tongguan liquid, Baihe and Kuh-seng enema, Kuh-seng Huaihua mixture	SASP, Mezalazine	9 RCTs
60	Wu N2019	Decoction	Systematic evaluation of the efficacy and safety of Huangqin Decoction in UC.	*n* = 777	Huangqin Decoction	SASP, Mezalazine, Probiotics	10 RCTs
61	Peng JF2019	Decoction, Chinese patent medicine	To evaluate the therapeutic effect of TCM retention enema in treating UC.	*n* = 2477	Colon An Liquid, Yuanxing Changan Liquid, Shengji Powder, Xihuang Mixture, Kuijie Powder, Huanglian Decoction, Xilei Powder, Kangfuxin Liquid, Pearl Guchang Powder, Jiaodai Decoction	SASP, Mesalazine	28 RCTs
62	Fan 2019	Decoction	To evaluate the efficacy of Gegen Qinlian Decoction for UC.	*n* = 2028	Gegen Qinlian Decoction, Gegen Qinlian Wutan Decoction	Olsalazine, Sulfasalazine, Methalazine, *B. subtilis*, hydrocortisone, sodium succinate	22 RCTs
63	Chi RT2019	NR	To evaluate the clinical efficacy and safety of Shenqi Baizhu Powder combined with mesalazine in the treatment for UC	*n* = 2380	Shenling Baizhu Powder	Methalazine	17 RCTs
64	Tang XJ2020	NR	To evaluate the clinical efficacy of Jianpi qingrehuoxue therapy for UC	*n* = 2374	Chinese herbal medicines guided by Jianpi qingrehuoxue therapy	Methalazine, SASP, live binary *B. subtilis*	28 RCTs
65	Liao ZW2020	Chinese patent medicine	To assess the efficacy and safety of Danshen Injection in adjuvant treatment of UC	*n* = 1102	Danshen Injection	Methalazine, SASP, live binary *B. subtilis*	12 RCTs
66	Long TJ2020	Decoction	To evaluate the efficacy of oral Chinese herbal compound on UC with damp-heat syndrome of large intestine in RCTs	*n* = 378	Gegen Qinlian decoction, Yigong powder, Shaoqi chunpi decoction, Qufeng ningkui decoction, Hongteng baijiang baitouweng decoction, Baitouweng decoction	Methalazine, SASP	6 RCTs
67	Long CW2021	Decoction, Chinese patent medicine	To evaluate the clinical efficacy of Sishen pill plus or reduce or combined with retention enema in the treatment of UC	*n* = 680	Sishen Pill	Methalazine, SASP, Hydrocortisone	9 RCTs
68	Bo HJ2020	Decoction	To evaluate the therapeutic effect of Wumei pill in treating UC	*n* = 1219	Wumei Pill	Methalazine, SASP	16 RCTs
69	Li PF2020	Decoction	To evaluate the clinical efficacy and safety of Chinese herbal compound enema in the treatment of UC	*n* = 1597	Baitouweng decoction, lianbei mixture, Yuyang liquid, Qinjiao Cangzhu decoction, Xileisan, Fufang Juhua granule, Changyu enema prescription, Changyu enema prescription, Qingre Zhixue decoction, Fufang Huangbai liquid, Huangqi decoction, kuijieqing, Qingre Qushi decoction, Sanhuang Decoction	Methalazine, SASP	17 RCTs
70	Tan GZ2020	NR	To evaluate the clinical efficacy and safety of TCM enema combined with mesalazine in treating UC	*n* = 2272	NR	NR	29 RCTs
71	Hu QH2021	Decoction	To analyze the clinical efficacy of TCM in the treatment of UC with damp-heated syndrome of large intestine in using Meta.	*n* = 695	Baitouweng decoction, Banxia Xiexin decoction, Changqingshu decoction, Changyuning granule, Gexian decoction, Jianpi Guchang decoction, Jiechang decoction, Qingchang Huashi decoction, Kuijie decoction	Methalazine, SASP	9 RCTs
72	Yan ZX 2021	Decoction	To assess the effcacy and safety of retention enema with TCM for UC	*n* = 1392	Tin-like powder, Huangkui Lianchang prescription, Huangkui Lianchang prescription, Baishao Qiwu Granules, Baitouweng and Lizhong decoctions, Hongteng decoction, Hongteng Decoction, Hongteng decoction, Buzhong Yiqi Decoction, Buzhong Yiqi decoction, kuijie decoction, Wubeizi powder, Kuiyangning decoction and some self-made Chinese herbal decoction	Tin-like powder, Mezalazine, SASP, 4-ASA, Gentamycin, Metronidazole sodium chloride injection, Prednisolone, Dexamethasone,	17 RCTs
73	Yuan H2020	Decoction	To evaluate the efficacy and safety of Six Gentlemen Decoction intake in the treatment of UC	*n* = 614	Six Gentlemen Decoction	Methalazine, Budesonide, Spleen Yi Chang Pill	7 RCTs

1. *UC* Ulcerative colitis 2. *SASP* salazosulfapyridine 3.*NR* not reported 4. *5-ASA* 5-aminosalicylicacid 5.*DAI* DNA-dependent activator of IFN-regulatory factors 6. *IL* innammatory factors levels of interlekin 7. *ESR* erythrocyte sedimentation rate 8.*TNF-α* tumor necrosis factor α 9.*CRP* C-reaction protein 10. *IgA* immunoglobulin A 11.*IgM* immunoglobulin M 12.*IgG* immunoglobulin G

## Appendix 2

**Table 3:** Outcomes of the included systematic reviews

No.	Study ID (first author, year)	Outcomes (+: for positive, −: for negative)
1	Gong Y2014	Total effectiveness (+) **RR 1.22 [1.15, 1.30]**, Adverse reaction rate (+)
2	Li WH2013	Total effectiveness (+) **OR 5.50 [3.74, 8.08]**, Cure rate (+) **OR 3.26 [2.44, 4.35]**, Adverse reaction rate (+)
3	Zuo HB2013	Total effectiveness (+) **RR 1.75 [1.24, 2.48]**, Adverse reaction rate (+), Clinical symptoms (+), Electron enteroscopy results (+)
4	Pei QW2012	Total effectiveness (+) **OR 3.87 [2.47, 6.05]**, Adverse reaction rate (+)
5	Wen Y2017	Clinical effectiveness (+) **RR 1.55 [1.39, 1.72]**, Adverse reaction rate (+), DAI (+), TNF-a (+), IL-17 (+), CRP (+)
6	Chen K2016	Total effectiveness (+) **OR 3.30 [2.25, 4.82]**, significant effectiveness (+) **OR 2.02 [1.54, 2.65]**, inefficiency rate (+) **OR 0.30 [0.21, 0.44]**
7	Lu2017	Inefficiency rate (+) **OR 0.26 [0.20, 0.35]**, recurrence rate (+), DAI (+), TNF-a (+), IL-17 (+), IL-23 (+), CRP (+)
8	Wang XY2017	Total effectiveness (+) **OR 4.44 [2.65, 7.44]**, Cure rate (+) **OR 1.72 [1.08, 2.75]**, Adverse reaction rate (+), Recurrence rate (+)
9	Wei Y2018	Clinical effectiveness (+) **OR 6.03 [1.95, 16.46]**
10	Zhang LH2018	Total effectiveness (+) **OR 4.32 [2.55, 7.31]**, Adverse reaction rate (+), Recurrence rate (+)
11	Li HB2017	Total effectiveness (+) **OR 4.69 [3.00, 7.34]**, Adverse reaction rate (−)
12	Li HB2018	Total effectiveness (+) **OR 2.16 [1.28, 3.63]**, Adverse reaction rate (−), TCM syndrome (−), mucosal lesion (+)
13	Huang FMZ2014	Total effectiveness (+) **RR 1.20 [1.15, 1.26]**, Recurrence rate (+), DAI (+), TCM syndrome (+), IgA* (+), IgM* (+), IgG* (+), Symptom relief time (+), stool occult blood (+), performance of colonoscopy (+)
14	Li HB-a2018	Total effectiveness (+) **OR 3.12 [2.11, 4.60]**, Adverse reaction rate (+), abdominal pain (+), diarrhea (−), pus and blood stool (−)
15	Liu G2011	Total effectiveness (+) **OR 0.18 [0.11, 0.32]**
16	Gu SZ2018	Total effectiveness (+) **RR 1.17 [1.12, 1.21]**, Adverse reaction rate (−), TCM syndrome (+), DAI (+)
17	Xiong AQ2011	Clinical effectiveness (+) **OR 3.71 [2.26, 6.10]**
18	Lv C2014	Total effectiveness (+) **RR 1.20 [1.13, 1.28]**, Adverse reaction rate (+), Recurrence rate (−), IL-13 (+), IL-8 (−), ESR (−), CRP (−), TCM syndrome (+), IgG (+), abdominal pain (+), diarrhea (+), pus and blood stool (+), Geboes (+), Mucosal biopsy score (−), performance of colonoscopy (+), Time of bellyache disappearance (+), Time of diarrhea disappearance (−)
19	Wang DY2011	Clinical effectiveness (+) **RR 1.26 [1.11, 1.43]**, Adverse reaction rate (+), Recurrence rate (+), pus and blood stool (−), performance of colonoscopy (+)
20	Liu TW2016	Total effectiveness (+) **RR 1.20 [1.14, 1.27]**, Adverse reaction rate (−)
21	Yang L2017	Clinical effectiveness (+) **RR 1.31 [1.19, 1.44]**, IL-6 (+), IL-8 (+), performance of colonoscopy (+), TCM syndrome (+),
22	Zhang WN2017	Total effectiveness (+) **RR 1.23 [1.15, 1.32]**
23	Chen F2012	Clinical effectiveness (+) **RR 1.27 [1.21, 1.34]**, Recurrence rate (+), performance of colonoscopy (+)
24	Yan SG2013	Total effectiveness (+) **OR 4.18 [2.95, 5.91]**, cure rate (+) **OR 2.86 [2.17, 3.76]**, Recurrence rate (+)
25	Xiong J2008	Clinical effectiveness (+) **OR 4.19 [2.89, 6.07]**, cure rate (+) **OR 3.12 [2.34, 4.15]**, Recurrence rate (+)
26	Chen MY2018	Total effectiveness (−) **RR 0.99 [0.91, 1.08]**, Adverse reaction rate (+), DAI (−), performance of colonoscopy (+)
27	Chen MY2018-a	Total effectiveness (+) **RR 1.20 [1.13, 1.26]**, Adverse reaction rate (−), Recurrence rate (+), performance of colonoscopy (+), Time of bellyache disappearance (+), Time of diarrhea disappearance (−), Time of hematochezia disappearance (+)
28	Cui DJ2012	Clinical effectiveness (−) **RR 0.97 [0.70, 1.35]**, Adverse reaction rate (−),
29	Ma XM2012	Total effectiveness (+) **OR 5.29 [3.67, 7.63]**, cure rate (+) **OR 3.65 [2.61, 5.12]**, Adverse reaction rate (+), DAI (+), performance of colonoscopy (+), abdominal pain (+), diarrhea (+), pus and blood stool (+)
30	Lai YL2013	Total effectiveness (+) **RR 1.14 [1.06, 1.23]**, cure rate (+) **RR 1.54 [1.18, 2.00]**, Adverse reaction rate (+)
31	Zhu JB2016	Total effectiveness (+) **OR 5.20 [2.63, 10.29]**
32	Huang ZB2014	Total effectiveness (+) **OR 4.05 [2.98, 5.50]**, cure rate (+) **OR 3.24 [2.57, 4.09]**, Adverse reaction rate (+)
33	Qi J2016	Total effectiveness (+) **OR 4.97 [1.73, 14.33]**, cure rate (+) **OR 3.51 [1.92, 6.42]**
34	luo Y2012	Total effectiveness (+)
35	Xu P2015	Total effectiveness (+) **OR 4.28 [3.16, 5.79]**, Adverse reaction rate (+), DAI (+), IL-6 (−), performance of colonoscopy (+), Time of bellyache disappearance (+), Time of diarrhea disappearance (+), Time of hematochezia disappearance (+), Time of fever disappearance (+)
36	Gong YD2012	Clinical effectiveness (+) **OR 4.54 [3.29, 6.18]**, DAI (+), ESR (+), performance of colonoscopy (+), IgA (−), IgM (+), IgG (−), Whole blood viscosity score (−), plasma viscosity (+), abdominal pain (+), diarrhea (+), pus and blood stool (+), tenesmus (+)
37	Ma DZ2015	Clinical effectiveness (+) **OR 4.2 [2.72, 6.49]**
38	Huang SG2010	Clinical effectiveness (+) **OR 6.67 [4.22, 10.53]**
39	Ni XX2019	Total effectiveness (+) **RR 1.20 [1.15, 1.25]**, Adverse reaction rate (+), Recurrence rate (+), performance of colonoscopy (+), TCM syndrome (+)
40	Jiang T2006	Clinical effectiveness (+), Total effectiveness (+) **OR 0.24 [0.14, 0.39]**, Adverse reaction rate (+)
41	Zhu XG2012	Total effectiveness (+) **OR 6.10 [4.33, 8.60]**
42	You WF2017	Clinical effectiveness (+) **OR 6.34 [3.97, 10.14]**
43	Cui DJ-a2012	Total effectiveness (+) **RR 1.16 [1.07, 1.25]**, Adverse reaction rate (+), Recurrence rate (+)
44	Wu Zl2017	Total effectiveness (+) **RR 1.25 [1.19, 1.31]**, Adverse reaction rate (+), Recurrence rate (+), ESR (+)
45	Huang HJ2012	Total effectiveness (+) **OR 13.36 [4.90, 36.46]**, Cure rate (+) **OR 4.55 [2.90, 7.14]**
46	Zha AS2015	Total effectiveness (+) **RR 1.248 [1.187, 1.313]**, Adverse reaction rate (+)
47	Hou LW2017	Clinical effectiveness (+), Total effectiveness (+) **RR 1.24 [1.18, 1.30]**, Adverse reaction rate (+), Recurrence rate (+), performance of colonoscopy (+)
48	Hou LW2015	Clinical effectiveness (+), Total effectiveness (+) **RR 1.32 [1.23, 1.41]**, Cure rate (+) **RR 1.91 [1.35, 2.70]**, Adverse reaction rate (+), Recurrence rate (+), performance of colonoscopy (+), TCM syndrome (+)
49	Gan YK2015	Clinical effectiveness (+) **OR 3.36 [1.96, 5.76]**
50	Zhu JM2011	Clinical effectiveness (+) **RR 1.13 [1.04, 1.23]**, performance of colonoscopy (−), TCM syndrome (+)
51	Wang DY2013	Clinical effectiveness (+) **OR 5.16 [3.35, 7.95]**, Cure rate (+) **OR 2.9 [2.10, 3.98]**, Adverse reaction rate (+), Recurrence rate (+)
52	Wang Y2018	Bifidobacterium level (+), Lactobacillus level (+), Enterococcus level (+), *E. coli* level (+)
53	He M2007	Clinical effectiveness (+), Total effectiveness (+) **OR 0.26 [0.16, 0.42]**
54	Pei QW2013	Total effectiveness (+) **OR 5.06 [3.41, 7.52]**, Adverse reaction rate (+), performance of colonoscopy (+), TCM syndrome (+)
55	Yang AX2006	Clinical effectiveness (+), Total effectiveness (+) **OR 6.60 [4.60, 9.47]**
56	Zhu L2012	Clinical effectiveness (+) **RR 1.17 [1.10, 1.25]**
57	Jia JW2019	Total effectiveness (+) **OR 4.99 [3.48, 7.14]**, IL-6 (+), IL-10 (+), CRP (+), Mucosal biopsy score (+)
58	Li L2019	Adverse reaction rate (+), Clinical efficacy (+) **RR 4.93 [3.35, 7.26]**
59	Chen MJ2019	Total effectiveness (+) **OR 0.13 [0.08, 0.18]**, Adverse reaction rate (+)
60	Wu N2019	Total effectiveness (+) **RR 1.23 [1.14, 1.31]**,Adverse reaction rate (+), IL-6 (+), TNF-α (+), IgA (+), IgG (+)
61	Peng JF2019	Total effectiveness (+) **RR 1.17 [1.13, 1.21]**, Recurrence rate (+), Adverse reaction rate (+)
62	Fan 2019	Total effectiveness (+) **RR 1.21 [1.12, 1.31]**, Recurrence rate (+), Adverse reaction rate (+),performance of colonoscopy (+)
63	Chi RT2019	Total effectiveness (+) **OR 3.35 [2.45, 4.60]**, DAI (+),Adverse reaction rate (−), TCM syndrome (+), TNF-α (+),IL-17 (+), IL-23 (+), ESR (+), CRP (+)
64	Tang XJ2020	Total effectiveness (+) **RR 1.18 [1.14, 1.23]**
65	Liao ZW2020	Total effectiveness (+) **RR 1.23 [1.16, 1.29]**, Recurrence rate (+), Adverse reaction rate (−), TNF-α (+), IL-6 (+), IL-8 (+), MPV (+), PLT (+), FIB(+)
66	Long TJ2020	Total effectiveness (+) **OR 3.84 [2.07, 7.13]**, TCM syndrome (+), Mucosal biopsy score (+)
67	Long CW2021	Total effectiveness (+) **RR 1.19 [1.07, 1.31]**, Cure rate (+) **RR 1.72 [1.44, 2.06]**, Adverse reaction rate (+), ESR (−), CRP (+)
68	Bo HJ2020	Total effectiveness (+) **RR 1.24 [1.18, 1.30]**,Recurrence rate (+), Adverse reaction rate (+), Efficacy of mucosal lesions (+)
69	Li PF2020	Total effectiveness (+) **RR 1.31 [1.25, 1.37]**, Recurrence rate (+), Mayo score (+), Adverse reaction rate (+), TCM syndrome (+), Mucosal biopsy score (+)
70	Tan GZ2020	Total effectiveness (+) **OR 4.90 [3.75, 6.41]**, Adverse reaction rate (+), Efficacy of mucosal lesions (+)
71	Hu QH2021	Clinical effectiveness (+) **RR 1.20 [1.12, 1.29]**, TCM syndrome (+)
72	Yan ZX 2021	Clinical effectiveness (+) **OR 3.87 [2.71, 5.51]**, Recurrence rate (+), Efficacy of mucosal lesions (+)
73	Yuan H2020	Clinical effectiveness (+) **OR 0.22 [0.13, 0.39]**, Adverse reaction rate (+)

1.*DAI* DNA-dependent activator of IFN-regulatory factors 2. *IL* innammatory factors levels of interlekin 3. *ESR* erythrocyte sedimentation rate

4.*CRP* C-reaction protein 5. *IgA* immunoglobulin A 6. *IgM* immunoglobulin M 7. *IgG* immunoglobulin G 7.*MPV* meanplateletvolume 8.*PLT* platelet 9.*FIB* fibrinogen

## Abbreviations

AGA: American gastroenterology association
AMSTAR 2: A measurement tool to assess systematic reviews 2
BSG: British society of gastroenterology
CSGE: Chinese Society of gastroenterology
CD: Crohn’s disease
CRP: C-reaction protein
DAI: DNA-dependent activator of IFN-regulatory factors
ESR: Erythrocyte sedimentation rate
IBD: Inflammatory bowel disease
IgA: Immunoglobulin A
IgM: Immunoglobulin M
IgG: Immunoglobulin G
IL: Inflammatory factors levels of interleukin
PICOS: Participants, intervention, control, outcome, study design
SRs: Systematic reviews
TCM: Traditional Chinese medicine
UC: Ulcerative colitis
UC-CRC: UC-related colorectal cancer
